# Neurophysiological Examinations as Adjunctive Tool to Imaging Techniques in Spontaneous Intracerebral Hemorrhage: IRONHEART Study

**DOI:** 10.3389/fneur.2021.757078

**Published:** 2021-10-27

**Authors:** Klára Fekete, Judit Tóth, László Horváth, Sándor Márton, Máté Héja, László Csiba, Tamás Árokszállási, Zsuzsa Bagoly, Dóra Sulina, István Fekete

**Affiliations:** ^1^Department of Neurology, Faculty of Medicine, University of Debrecen, Debrecen, Hungary; ^2^Department of Radiology, Faculty of Medicine, University of Debrecen, Debrecen, Hungary; ^3^Department of Pharmaceutical Surveillance and Economics, Faculty of Pharmacy, University of Debrecen, Debrecen, Hungary; ^4^Institute of Political Science and Sociology, Faculty of Arts, University of Debrecen, Debrecen, Hungary; ^5^MTA-DE Cerebrovascular and Neurodegenerative Research Group, University of Debrecen, Debrecen, Hungary; ^6^Division of Clinical Laboratory Sciences, Department of Laboratory Medicine, Faculty of Medicine, University of Debrecen, Debrecen, Hungary

**Keywords:** hemorrhagic stroke, EEG, TMS, outcome, neurophysiological examination

## Abstract

**Introduction:** Intracerebral hemorrhage (ICH) is a devastating disease, which may lead to severe disability or even death. Although many factors may influence the outcome, neurophysiological examinations might also play a role in its course. Our aim was to examine whether the findings of electroencephalography (EEG) and transcranial magnetic stimulation (TMS) can predict the prognosis of these patients.

**Methods:** Between June 1 2017 and June 15 2021, 116 consecutive patients with ICH were enrolled prospectively in our observational study. Clinical examinations and non-Contrast computed tomography (NCCT) scan were done on admission for ICH; follow-up NCCT scans were taken at 14 ± 2 days and at 3 months ± 7 days after stroke onset. EEG and TMS examinations were also carried out.

**Results:** Of the patients in the study, 65.5% were male, and the mean age of the study population was 70 years. Most patients had a history of hypertension, 50.8% of whom had been untreated. In almost 20% of the cases, excessive hypertension was measured on admission, accompanied with >10 mmol/L blood glucose level, whereas their Glasgow Coma Scale was 12 on average. Presence of blood in the ventricles or subarachnoid space and high blood and perihematomal volumes meant poor prognosis. Pathological EEG was prognostic of a worse outcome. With TMS examination at 14 days, it might be possible to estimate outcome in a univariate model and the absence, or reduction of the amplitude of the motor evoked potentials was associated with poor prognosis.

**Conclusion:** Together with the clinical symptoms, the volume of bleeding, perihematomal edema (or their combined volume), and neurophysiological examinations like EEG and TMS play an important role in the neurological outcome of patients with ICH. This might affect the patients' rehabilitation plans in the future, since with the help of the examinations the subset of patients with potential for recovery could be identified.

## Introduction

Similarly to all developed countries, Hungary also struggles with the burden of stroke. Although non-Traumatic intracerebral hemorrhage (ICH) affects 1 in 10 patients, case fatality and severe disability are very high among them (1–3). The incidence and the 30-day mortality of ICH are high (~40%), in elderly patients (4, 5). In addition, hemorrhage in the brain stem, intraventricular dissection, hematoma volume ≥30 cm^3^ and Glasgow Coma Scale (GCS) score < 5 are also poor prognostic factors (5, 6).

According to Gebel et al. (7), the absolute edema volume was not independently associated with 30-day mortality. They investigated the relative edema (edema volume divided by hematoma volume), and it was strongly predictive of functional outcome.

Recently, we published that there might be another useful predictor of ICH outcome. A conventional *in vitro* clot lysis assay (CLA) and a modified CLA (mCLA)—including cell-free DNA and histones—were performed from stored platelet-free plasma, taken on admission. Parameters of mCLA correlated with ICH bleeding volume (8).

ICH is not a single entity; 85% of the cases are due to cerebral small vessel disease, predominantly deep perforator arteriopathy (hypertensive or arteriosclerotic) and cerebral amyloid angiopathy, whereas the rest result from macrovascular causes (arteriovenous malformation, cavernoma, aneurysm, venous thrombosis) (5).

Neurophysiological examinations might also have potentials in predicting ICH outcomes; however, the prognostic value of electroencephalography (EEG) and motor evoked potentials (MEPs) in ICH outcome has not been extensively studied. Changes in the extent of bleeding and the onset and progression of brain edema can be monitored by using digital EEG and MEP techniques. In patients with larger hematomas of 30 mL or more, causing a shift of the midline structures, delta wave activity appears over both hemispheres (9). The incidence of seizures after spontaneous ICH reportedly ranges from 2.8 to 18.7% (10). Provoking factors are related to hemorrhage volume, location, cortical involvement, and the severity of neurological deficits (11, 12). Seizures can be non-Convulsive, which remain undiscovered without an EEG examination (13).

Damage of the corticospinal tract can be examined with MEP elicited by transcranial magnetic stimulation (TMS). Based on the literature, the absence of MEP in the acute stage indicates poor recovery of muscle strength, whereas the presence of MEP in a completely hemiplegic patient predicted some recovery of motor function (14). The suppression of amplitude was more accurate than the prolongation of latency in predicting functional recovery. MEP monitoring of patients with ICH in the acute stage can predict the outcome of motor function (14–16).

According to the literature, it is not in line with the useful time of the TMS study (acute or subacute stage), and there is no follow-up study on which parameters are more characteristic of the assessment of functional prognosis.

The main goals of the follow-up neurophysiological study were to investigate (1) whether the EEG correlated with the improvement or deterioration of cerebral functions, comparing data with clinical parameters [National Institutes of Health Stroke Scale (NIHSS) score, modified Rankin Scale score] and cranial computed tomography (CT) findings (measured blood volume along with edema volume; (2) early detection of epileptiform discharges or non-Convulsive seizures to select patients who may benefit from early-initiated antiseizure drug treatment. In parallel with the EEGs, we performed TMS examinations to evaluate the prognosis and the probable effectiveness of further rehabilitation (17).

The main purpose was to find correlation between the volume of ICH—along with perihematomal edema, clinical signs, EEG, and magnetic evoked potential—and functional outcome.

## Methods

### Patients

Between June 1 2017 and June 15 2021, 116 consecutive patients with ICH were enrolled prospectively in our observational study, conducted at the Stroke Centre (Stroke Unit, Department of Neurology, University of Debrecen, Hungary) in the GINOP “IRONHEART Study.” Hereby we focus on special issues only, in association with another study, since the detailed protocol was published in it quite recently (17).

Inclusion criteria were as follows: patients older than 18 years with acute non-Traumatic ICH, verified with non-Contrast CT (NCCT) scan, not meeting exclusion criteria, and written informed consent.

Exclusion criteria included the presence of cerebral aneurysm, arteriovenous malformation, traumatic intracerebral bleeding (epidural or subdural hemorrhage, brain contusion), malignancy, hemorrhagic brain metastasis, severe hepatic and renal insufficiency, hemorrhagic diathesis, and severe acute respiratory syndrome coronavirus 2 (SARS-CoV-2) infection on hospital admission or during follow-up.

**Figure 1:**
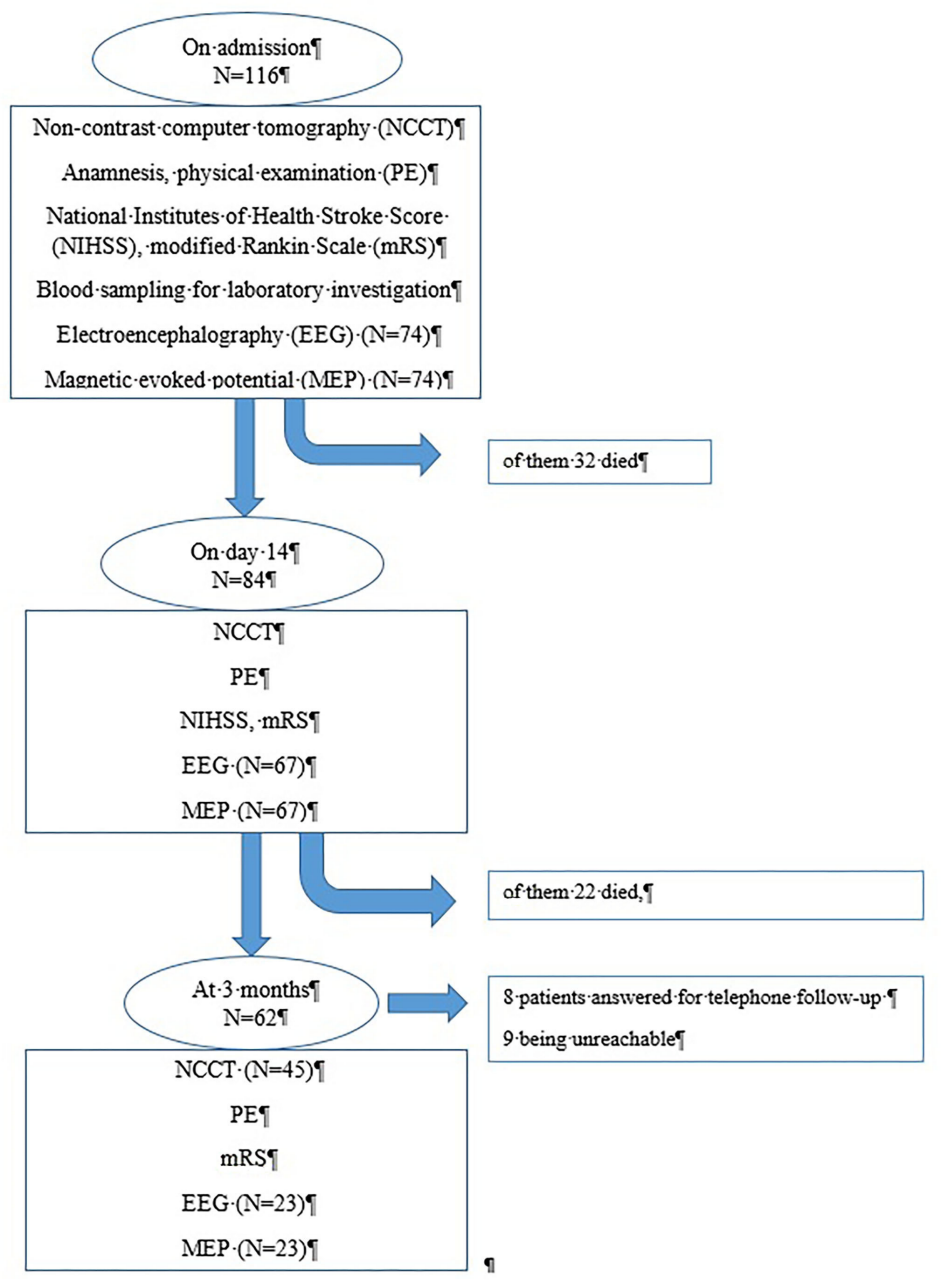
Flowchart: examination of the patients admitted for brain hemorrhage.

A flowchart of examinations is summarized in Figure 1.

On admission, an NCCT scan was done in addition to a physical examination, and when necessary, contrast-enhanced CT or CT angiography was performed to exclude secondary causes of ICH. Follow-up NCCT scans were performed at 14 ± 2 days and at 3 months ± 7 days after the onset of stroke.

All CT scans were performed with a 64-slice CT (type: General Electric Lightspeed VCT, software: 11HW12.5). Slice thickness was 2.5 mm infratentorially and 5 mm supratentorially. CT images were analyzed simultaneously by three senior independent radiologists trained in neuroradiology, and a detailed list of radiological data, such as location and volume of bleeding, extent of perihematomal edema, bilateral ventricular/bilateral cortical diameter, degree of midline shift, involvement of pyramidal tract, and presence of intraventricular or subarachnoid component, were recorded (18). The volume of intraventricular hemorrhage (IVH) was not measured, but the location of the blood in the ventricles was analyzed in every patient. Manual CT volumetry was performed by tracing the focal hyperdense (blood) and perifocal hypodense area surrounding the hemorrhage (edema) on each slice. Hypodensity attributable to microangiopathy was omitted as far as possible by comparison with the contralateral hemisphere. Total lesion volume was calculated by multiplying the traced area (ROI, cm^2^) with slice thickness and adding up the results (19–21). All tracings were performed by one radiologist (17).

The time at symptom onset, baseline characteristics (age, sex, cerebrovascular risk factors, history of cerebrovascular and cardiovascular diseases, previous medications) were recorded on admission.

GCS was used to assess the level of consciousness. Categories were formed as follows: ≤8 points (severe disturbance, usually means coma), 9 to 12 points (moderate disturbance), and 13 to 15 points (minor disturbance); in case of 15 points, the patient is fully alert.

Stroke severity was given according to the NIHSS on admission, 14 ± 2 days and 3 months ± 7 days after the onset of hemorrhagic stroke (22). mRS was estimated by two neurologists of the study (including physical examination) on admission (in acute stage), at 14 ± 2 days, at discharge (the time when the patient left the hospital, or died in hospital), and for evaluating long-term outcomes (3 months after the onset of stroke) (23).

### Laboratory Examinations

Currently, only on-admission blood glucose levels and INR values were analyzed. Detailed hemostasis, renal function, white blood cells, hemoglobin, hematocrit, and platelet count data were described in our previous article (8).

### Outcomes

The following primary outcomes were defined: (1) case fatality at discharge, (2) 3-month mortality, (3) long-term outcome at 3 months ± 7 days after the onset of stroke: mRS 0–2 is defined as favorable long-term outcome. If a patient's clinical status was very severe and/or they were in an unstable condition and therefore could not be transferred, neurophysiological examinations could not be done. If the patients did not come back for their 3 month follow-up examinations, we arranged for a telephone consultation in order to gather information of mRS. The study design was developed in accordance with the guiding principles of the Declaration of Helsinki and was approved by the Institutional Ethics Committee of the University of Debrecen and the Ethics Committee of the National Medical Research Council. All patients or their relatives provided written informed consent. A general consent form encompassed the consent for blood sampling, imaging studies, and neurophysiological examinations. All patient data were treated anonymously.

### Neurophysiological Examinations

The patients underwent digital EEG and TMS examinations. They were examined on three occasions using the above neurophysiological examinations within 24 to 48 h after admission, at 14 days ± 2 days and 3 months ± 7 days after the event. EEGs and MEPs were analyzed by two senior clinical neurophysiologists participating in the study. Both examinations were recorded in the EEG Clinical Neurophysiological Laboratory of the Department of Neurology according to the guidelines of the International Federation of Clinical Neurophysiology.

### Electroencephalography

Scalp EEG was performed for 40 min. All EEGs were recorded with Brain Quick SD PLUS, System Plus Evolution software (Micromed S.p.A. Italy) in a semi-isolated room. Nineteen channels were used and the electrodes (silver-silver chloride) were placed according to the 10–20 System (19 standard scalp positions, earlobes against Fpz sampling reference) with the help of adhesive and conductive gel. Impedance was < 10 kOhms. The patients were awake (with the exception of patients with disturbance of consciousness), in a relaxed state with closed eyes in a semisupine position. The filter was set as follows: 0.1 and 33.6 Hz; sampling rate: 256 per s, online digitization: 12 bit. Two board-certified senior neurophysiologists evaluated the recordings. Artifacts [electromyogram (EMG), electrocardiogram (ECG), electrooculogram (EOG), sweating, and others] were minimized and corrected during the recording real time with the help of experienced, trained technicians. If needed, EMG, EOG, ECG electrodes could be used, and the artifacts were also filtered and ruled out after recording also if needed. Medications potentially interfering with the EEG finding were left out 24 h before the on-admission EEG, at 14 days and 3 months' examination 3 days before the EEG. The EEG was evaluated in the absence of patterns indicating drowsiness or sleeping. A digital EEG program accomplished the average frequency, amplitude analysis, and EEG mapping if quantitative EEG was needed. Beta wave was determined if the frequency was 13 to 30 Hz (<30 μV), alpha wave between 8 and 13 Hz (30–50 μV), theta wave between 4 and 7 Hz (>50 μV), and delta wave in case the frequency was < 4 Hz (>100 μV).

In order to form groups, we used the terms as follows:

(1) Slow wave activity (focal or diffuse) was assessed in case of theta and delta background activity or when the frequency of the background rhythm was below the normal value for age and state. (2) Low-amplitude EEG activity was set in case the suppression of the background activity was < 10 μV (except of the frontal leads) or when there was a significant (50%) decrease in the amplitude compared to the previous recording. This was especially pronounced in case of large focal (hemispherical) bleeding compared with the contralateral side. (3) Epileptiform discharges were defined as spike, polyspikes, spike and slow wave complex, spike rhythm, sharp wave, and sharp and slow wave complex (24).

EEG worsening was defined always compared to the previous recordings as slowing of the background frequency (e.g., normal activity changed to focal or diffuse theta, delta activity, or the amplitude reduction was significant as defined above or suppressed vigorously). Appearance of the pathological EEG signs, such epileptiform discharges, also meant worsening.

### Transcranial Magnetic Stimulation

All examinations were done using Magstim 200 magnetic stimulator (9-cm-diameter coil, A face for right hemisphere, B face for left hemisphere) capable of generating 2T maximum field intensity. The MEPs were registered over the abductor digiti minimi muscle on both upper limbs and both tibial anterior muscles on the lower limbs with conventional cup electrodes. The responses were recorded with Dantec Keypoint (2012 Alpine Biomed ApS; the filters were set between 2 and 10 kHz. For stimulation, a 20% above-threshold and maximal stimulation output was used. Cortical (vertex/Cz using the International 10–20 System), cervical (CVII), and lumbar (LIV–V) regions were stimulated. For stimulation and facilitation, we used the technique published by Escudero et al. (25). The latencies and amplitudes were measured according to Rossini et al. (26). Total conduction time meant the time of the cortical stimulation to the registered answer in the muscle. Peripheral conduction time was measured by cervical and lumbar stimulation. Peripheral conduction time was subtracted from the total conduction time, in order to receive the central conduction time (CCT). All stimulations were repeated four times.

If despite the maximal output we failed to register an MEP, we repeated the examination four times again and only afterward declared as an absent answer. A pathological latency, amplitude, or CCT was declared if the value was not within ±2.5 SD of the normal database given for each parameter. Patients were selected after strict consideration of contraindications as described in the safety regulations of the TMS examination protocol (e.g., metallic implantations, pacemakers were excluding factors). The investigation was suitable to show the severity of damage to the corticospinal tract, which may correlate with the prognosis and outcome of rehabilitation even in a hemiplegic patient (12, 14).

### Statistical Analysis

For this study, descriptive statistical analysis was carried out using the SPSS for Windows 19.0 program suite (SPSS Inc. Chicago, IL, USA). Two-group analysis was assessed with Pearson χ^2^ test for categorical variables. For continuous variables, Mann-Whitney *U* test was used. The level of significance was set at *p* < 0.05. Logistic regression models were used to identify the independent predictors of 3 month disability. The analysis was performed with the multivariate general linear model. In the models, disability at 3 months (mRS > 2 was the dependent variable, and the factors found to be associated with outcome by univariate analyses were entered as confounding variables. The variables were excluded from the analysis one by one, and the variable with *p* > 0.05 and closest to 1.0 was removed, until all features left in the model had *p* > 0.05.

## Results

Baseline characteristics are summarized in Table 1. Among the 116 patients, significant male predominance (*p* < 0.05) was observed, and the majority of them had hypertension in their history, but more than half of the cases had been untreated. From the patients 25% suffered from diabetes mellitus, and 20.6% had cardiac failure or atrial fibrillation. The patients' mean age was 70 ± 11 years, the oldest and the youngest patients being 93 and 43 years old, respectively. The mean age of women was 73.4 years, whereas the relevant figure for men was 68.6 years.

**Table 1:** Baseline characteristics (main risk factors) of the patients (*p < 0.05; *N* = 116).

**Parameter**	**Value**
Age, mean ± SD (years)	70 ± 11
Gender: male/female	65.5%/34.5%*
Previous hypertension (not treated)	89% (50.8%)
Previous diabetes mellitus	25%
Previous ischemic stroke	13.7%
Previous hemorrhagic stroke	2.5%
Previous heart failure	7.7%
Previous atrial fibrillation	12.9%

**Figure 2:**
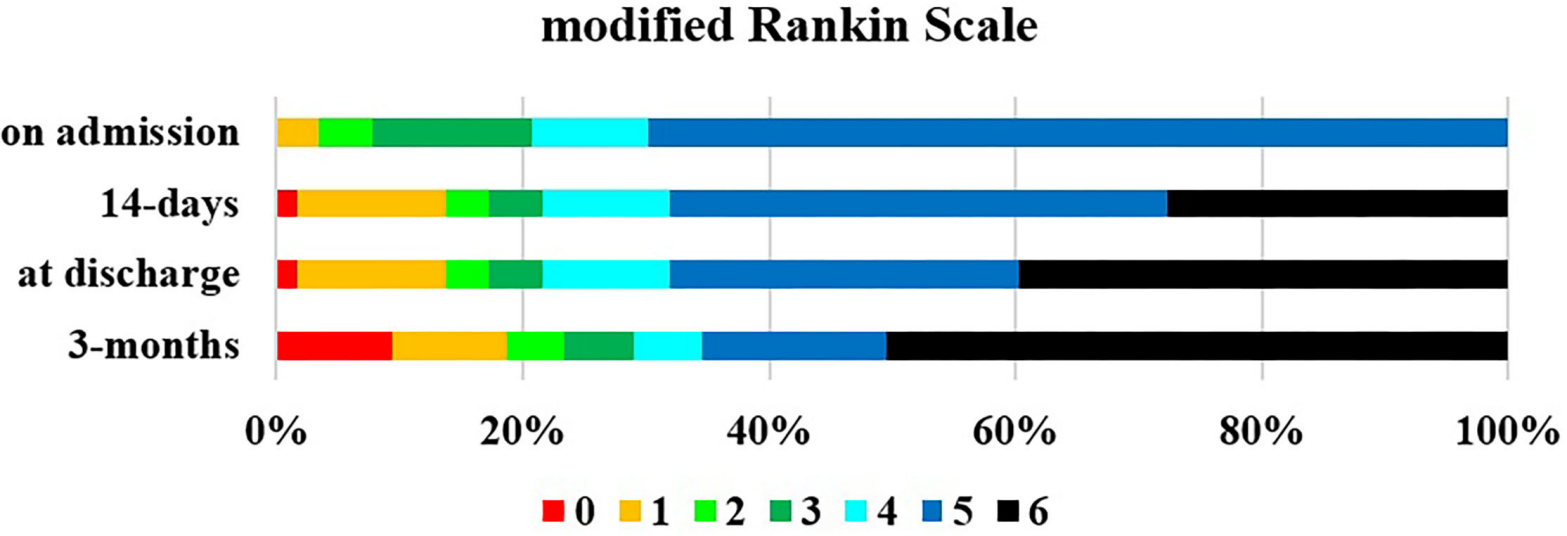
Outcome based on mRS on admission, at 14 ± 2 days, at discharge and at 3 months ± 7 days. Mean discharge time was 19.8 days (minimum 1 days, maximum 79 days).

On-admission parameters are summarized in Table 2. Systolic blood pressure was generally high, and in 19.85% of the patients, it measured extremely high (>200 mmHg). Similarly, 21.5% of the patients had hyperglycemia. More than one-third (39.6%) of the patients received antithrombotic therapy. On admission, stroke severity was moderate, but if the subsequent disability was regarded in terms of mRS, the outcome was much poorer, because most of the patients' scores were mRS 5 (Figure 2).

**Table 2:** Summary of on-admission parameters (*N* = 116), including scales (modified rankin scale on admission and glasgow coma scale, NIHSS scale).

**Parameter**	**Value**
Systolic blood pressure, mean ± SD *>200 mmHg*	180 ± 34 *19.8%*
Diastolic blood pressure	95.1 ± 21.5
Antithrombotic therapy *Antiplatelet therapy* *Anticoagulation* *Combined*	39.6% *19.8%* *12.9%* *6.8%*
On admission blood glucose level, mean ± SD (mmol/L) *>10 mmol/L*	8.6 ± 3.6 *21.5%*
On admission INR >1.7 (pts%)	11.2%
On admission NIHSS median (1st; 3rd quartile)	14.25 (8;19.25)
On admission modified Rankin Scale *0–2* *3–5 (5)* *6*	*7.7%* *92.7% (69.8%)* *0%*
Glasgow Coma Scale median ± SD *13–15* *9–12* *≤ 8*	12 ± 3 *52.5%* *34.5%* *13%*

*Important subgroup features are italicized*.

Imaging parameters are shown in Table 3, in which they are compared at different time points. Bleeding was more frequent in the left hemisphere (56%). Bleeding mostly affected the basal ganglia and/or thalamus (75%), lobes (15%), brainstem, and cerebellum (10%). If both the basal ganglia and thalamus or pons and the midbrain had been affected, a patient's prognosis was poor. Almost half of the CT scans revealed hydrocephalus, which improved over 3 months. The severity of our cases could be established by, e.g., the presence of blood in the prepontine cistern (7.7%), or by the intraventricular or subarachnoid propagation of the ICH in almost half of the patients. Midline shift was apparent on admission in 50% of the cases, and improvement could only be detected in 3 months. Based on the CT scans, a lesion of the pyramidal tract could be confirmed in the majority of the patients. However, no TMS abnormality was detected in 6% of the patients despite a possible pyramidal tract lesion shown in the CT scan.

**Table 3:** Summary of CT parameters on admission, 14 days and at 3 months.

**Parameter**	**Admission (*N* = 116)**	**14 days(*n* = 84)**	**3 months (*n* = 45)**	***p-*** **value**
Hydrocephalus (% pts)	49.1%	34.4%	17.6%	**<0.04**^**a**^ **<0.0005**^**b**^ **<0.048**^**c**^
Bilateral ventricular/bilateral cortical diameter mean ± SD (cm)	3.09 ± 0.88	3.8 ± 5.2	3.45 ± 0.7	<0.15^a^ **<0.015**^**b**^ <0.65^c^
Presence of blood in the prepontine cistern	7.7%	8.6%	0%	<0.88^a^ <0.15^b^ <0.14^c^
Right/left hemisphere	40.5%/56%	NA	NA	NA
Localization (basal ganglia + thalamus)	75%	NA	NA	NA
Volume of bleeding, mean ± SD (cm^3^)	26 ± 30.6	16.7 ± 21.1	3 ± 6.6	** <0.017**^**a**^ **<0.0001**^**b**^ **<0.0001**^**c**^
Volume of edema (cm^3^)mean ± SD	12.9 ± 15	24.6 ± 29	13.6 ± 24.8	** <0.0003**^**a**^ <0.83^a^ ** <0.033**^**c**^
Volume of bleeding + edema,mean ± SD (cm^3^)	39.1 ± 43	41.3 ± 45.1	18.3 ± 31.2	<0.73^a^ ** <0.0036**^**b**^ **<0.0028**^**c**^
Midline shift (mm)	3.1 ± 4.3	3 ± 3.8	0.5 ± 1.5	<0.86^a^ **<0.0001**^**b**^ **<0.0001**^**c**^
Intraventricular and subarachnoid appearance of bleeding (% pts)	47.4%	17.9%	4.4%	**<0.0001**^**a**^ **<0.0001**^**b**^ **<0.047**^**c**^
Pyramidal tract affected as revealed by CT	74.1%	57.1%	24.4%	**<0.012**^**a**^ **<0.0001**^**b**^ **<0.0006**^**c**^

*Correlation shown between parameters on admission and 14 days^a^, on admission and 3 months^b^, and 14 days and 3 months^c^, Significant values are in bold*.

We compared the volume of bleeding, volume of the edema, and the volume of bleeding plus edema together. The volume of bleeding gradually decreased, as expected. The volume of edema reached its maximum at 14 days and slowly returned to the on-admission value over 3 months. Thus, based on our neurophysiological examinations, the combined volume of the two fluids seemed to be a better predictor. The on-admission normal findings or mild abnormalities disappeared after 14 days and improved steadily over 3 months.

In our study intraventricular (IVH) or subarachnoid propagation of the ICH was found in almost half of the patients (47.4%) on admission, 17.6% at 14 days, and 3.4% at 3 months. Although we have not measured the IVH volume, similar to Graeb and colleagues' (27) publication, the location of the IVH was given. It was as follows: there was bleeding in unilateral/bilateral lateral ventricles in 5 patients, and 20 more patients also had blood also in their third and/or in the fourth ventricles; one patient had blood only in the fourth ventricle. Because of the high mortality and degradation of blood among survivors, on the second CT image, we could detect IVH in only seven patients, five of whom in the lateral ventricles and also the third or/and fourth ventricles. At 3 months, all patients with IVH had only a small amount of blood in the ventricles. The outcome given in mRS was significantly worse among patients with intraventricular bleeding than in those without it on admission, at 14 days and at 3 months; *p* = 0.002, *p* = 0.05, and *p* = 0.015, respectively.

Neurophysiological examinations could be carried out in 74 patients on admission. We demonstrate the difference in important parameters in Table 4 between patients undergoing EEG and TMS and patients who did not. Further results presented show the findings among those suitable for neurophysiological examinations.

**Table 4:** Comparison of important parameters and outcome of the groups with and without neurophysiological examinations.

**Parameter**	**Without neurophysiological examination**	**With neurophysiological examination**	***p-*** **value**
Age, mean ± SD (years)	71.9 ± 11.3	68.9 ± 11.3	0.2113
Hypertension [n (%)]	40 (95.2)	64 (86.5)	0.137
Diabetes mellitus [n (%)]	11 (26.2)	18 (24.3)	0.82
Previous stroke [n (%)]	6 (14.3)	10 (13.5)	0.91
Previous heart failure [n (%)]	4 (9.5)	5 (6.8)	0.6
Atrial fibrillation (%)	8 (19)	7(9.5)	0.14
Glucose, mean ± SD (mmol/L)	9.5 ± 3.4	8.2 ± 3.7	**0.011**
On admission GCSscore median (1st; 3rd quartile)	11 (6.5;13)	15(11;15)	**0.00001**
VP shunt or hematoma evacuation (%)	2 (4.7)	2 (2.7)	0.34
On admission mRS 1–2 3–4 5	0 (0%) 2 (4.8%) 40 (95.2%)	9 (12.2%) 24 (32.4%) 4 (55.4%)	** <0.00001**
14 day mRS 0–2 3–4 5 6	1 (2.4%) 4 (9.5%) 21 (50%) 16 (38.1%)	19 (25.7%) 13 (17.6%) 26 (35.1%) 16 (21.6%)	**0.0033**
At discharge mRS 0–2 3–4 5 6	1 (2.4%) 4 (9.5%) 7 (16.7%) 30 (71.4%)	19 (25.7%) 13 (17.6%) 26 (35.1%) 16 (21.6%)	** <0.00001**
3 month mRS 0–2 3–4 5 6 Unknown	4 (9.5%) 0 (4.8%) 2 (4.8%) 33 (78.6%) 3 (7.1%)	21 (28.4%) 12 (16.2%) 14 (18.9%) 21 (28.4%) 6 (8.1%)	**0.00018**
NIHSSS on admissionmedian (1st; 3rd quartile)	17 (13.25;22)	11 (6;18)	**0.00022**
NIHSSS at dischargemedian (1st; 3rd quartile)	43 (20.2;43)	11 (4;18)	**0.0001**
3 month mRS 0–2 3–4 5 6 Unknown	4 (9.5%) 0 (4.8%) 2 (4.8%) 33 (78.6%) 3 (7.1%)	21 (28.4%) 12 (16.2%) 14 (18.9%) 21 (28.4%) 6 (8.1%)	**0.00018**
NIHSSS on admissionmedian (1st; 3rd quartile)	17 (13.25;22)	11 (6;18)	**0.00022**
NIHSSS at dischargemedian (1st; 3rd quartile)	43 (20.2;43)	11 (4;18)	**0.0001**

*p < 0.05 was significant, marked in bold font*.

**Figure 3:**
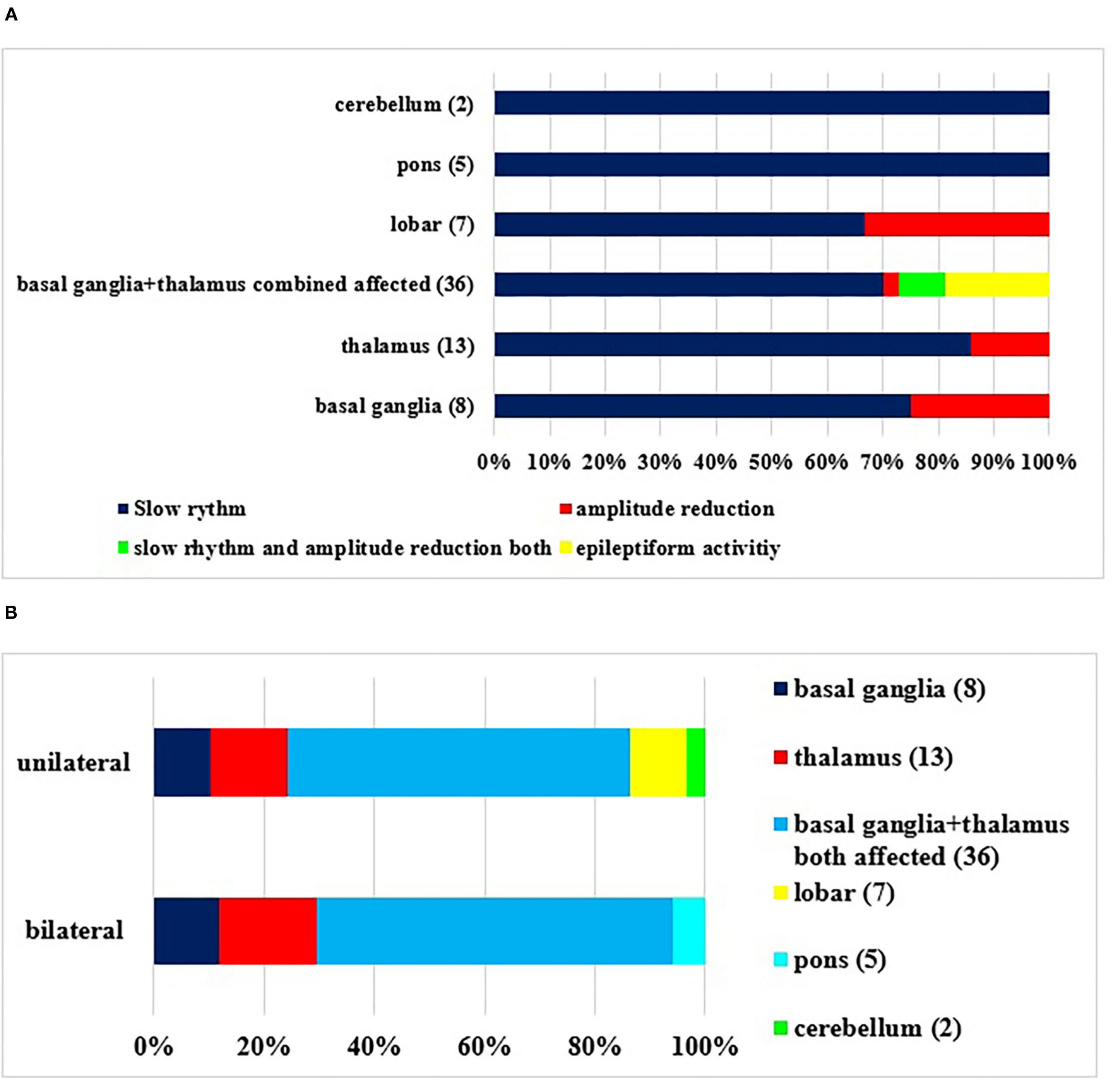
**(A,B)** Abnormalities in EEG during the examined period in accordance with the localization of primary source of the bleeding. The definitions of EEG alterations are given in detail in *Methods*. (Number of patients). There was a significant correlation between the type of EEG abnormality and localization (*p* < 0.05).

In Figure 3A, our *EEG* findings are presented according to the localization of the ICH. Slow rhythm was a much more common abnormality than amplitude reduction. Epileptiform discharges were detected when bleeding into the basal ganglia and that into the thalamus were combined, and only one patient had a clinical seizure within the examined period, but no one had clinical seizures during the recording based on the EEG and video monitoring.

Despite the unilateral hemorrhage, bilateral pathological findings (Figure 3B) were detected in the EEG, including all bleedings in the pons.

**Figure 4:**
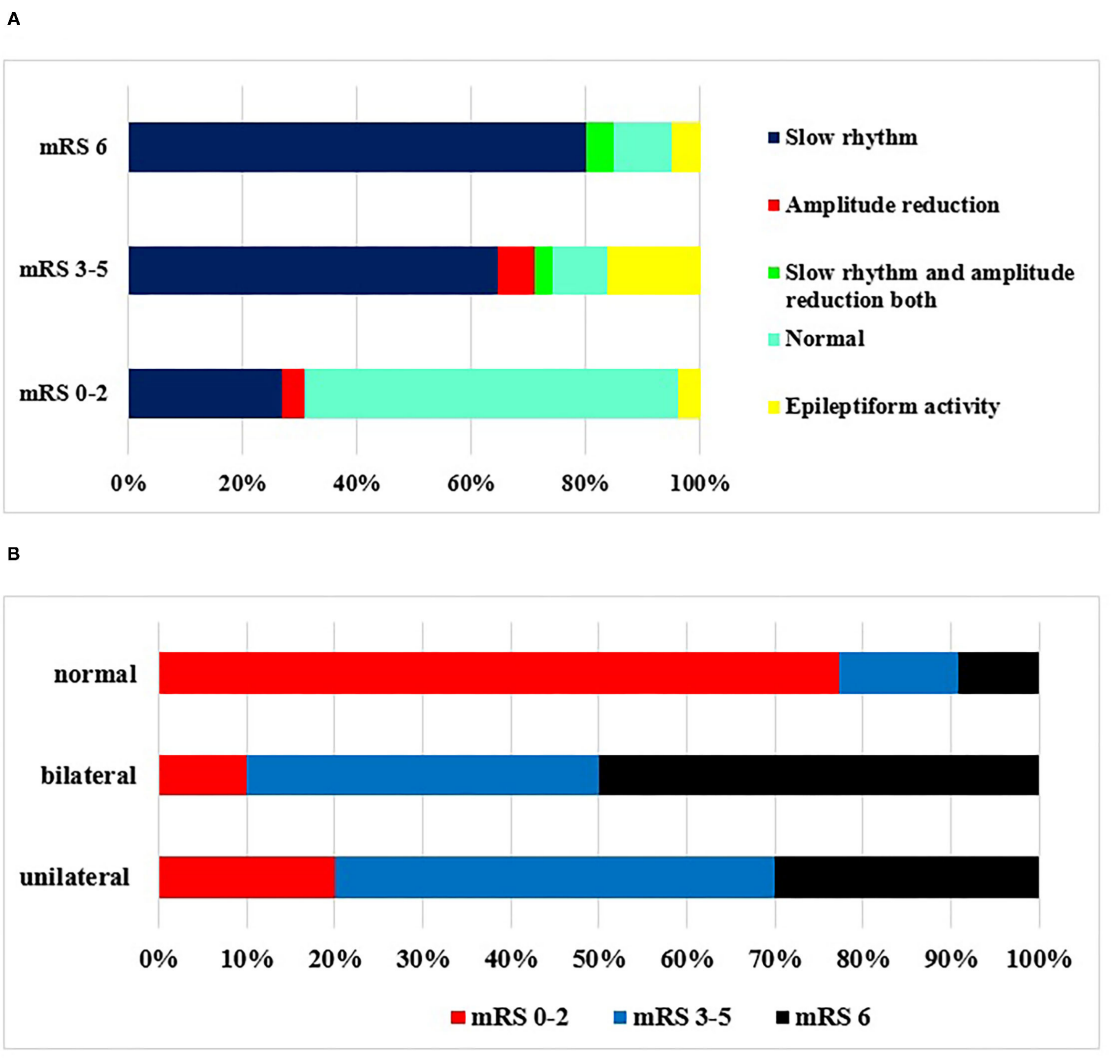
**(A,B)** EEG abnormalities during the examined period and outcome according to mRS at 3 months. Correlation was seen between poor outcome and presence of EEG abnormalities (*p* < 0.05). The definitions of EEG alterations are given in details in *Methods*. **(A)** Type of abnormality. **(B)** Unilateral/bilateral EEG abnormality.

If the EEG findings were set in another view (Figure 4A), the outcome was variable. One could conclude that a disabling 3 month status was probable when the on-admission or 14 day EEGs were pathological. It was true for both unilateral and bilateral abnormalities, although a bilateral one seemed to be a risk of a worse prognosis (Figure 4B). A normal EEG might prognosticate a good outcome.

**Figure 5:**
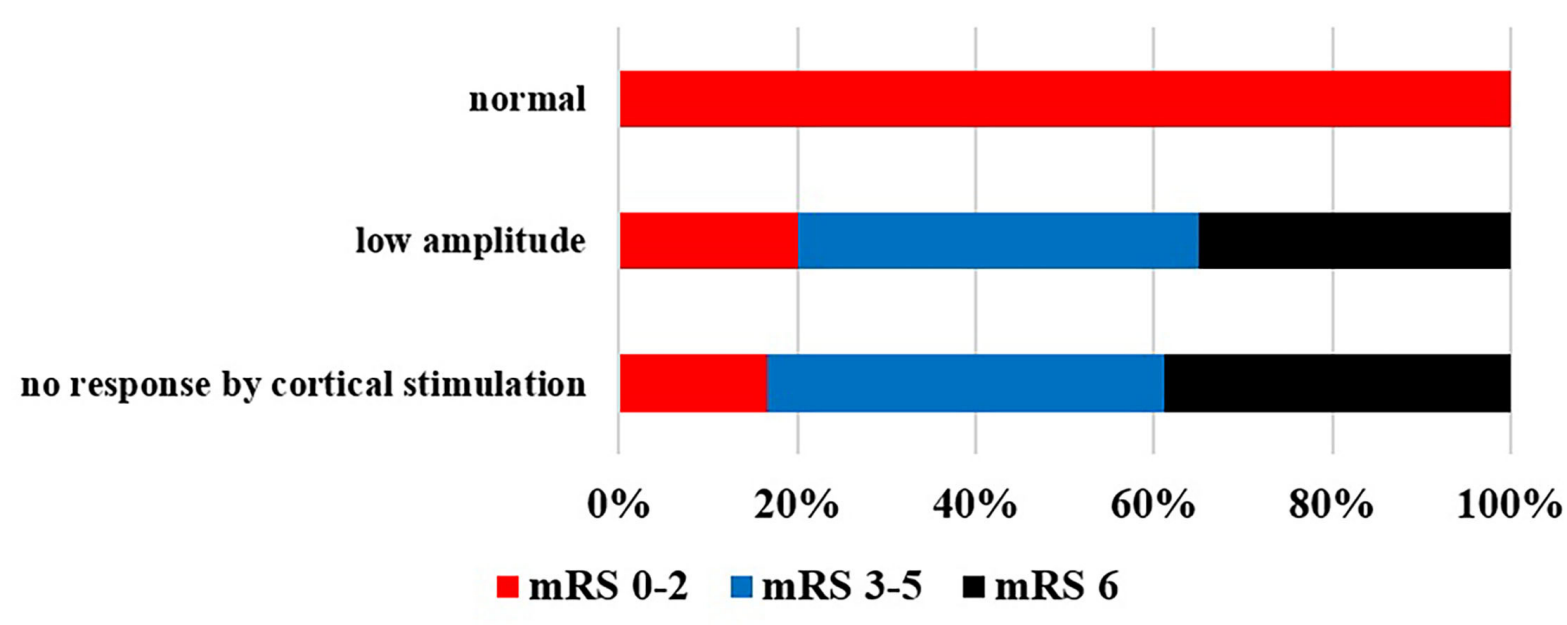
TMS findings during the examined period and outcome according to mRS at 3 months. Poor outcome was detected when MEP was absent or the amplitude reduced (*p* < 0.05).

TMS findings during the examined period and outcome according to mRS at 3 months are shown in Figure 5. Most commonly, no response to the affected sign could be detected, which meant poor prognosis. If a sign could be gained, the reduction in amplitude was the most prominent of the prognosis. In patients who were independent according to mRS (0–2), normal findings were detected. In case of abnormal registered answer on TMS examination, worse outcome was scored at 3 months (Figure 5). No TMS abnormality was detected in 6% of the patients, despite possible pyramidal tract lesions seen in the CT scan. In these cases, the pyramidal tract was just probably pushed aside by the volume of bleeding.

Altogether, if we wanted to find predictors of disability on admission, at 14 days and at 3 months different variables could be detected (Table 5). Age was only predictor of functional outcome at discharge [mean discharge time was 19.8 days (1;79)] and previous ischemic stroke at 3 months. Localization was an important predictor, just like blood in the subarachnoid space; ventricles were all predictors of mRS on admission and outcome at 14 days and 3 months. Bleeding, edema, and their combined volume all altered the disability significantly. Thirty-three percent of the patients received mannitol treatment, and 4% needed neurosurgical intervention. Two patients required intraventricular drain implantation because a blood clot blocked the circulation of the cerebrospinal fluid, whereas two patients with lobar hematoma underwent blood evacuation. If the CT scan revealed the pyramidal tract was affected, it had an impact on the admission and discharge mRS. A very important finding of ours—shown with two functional modalities (EEG, TMS) for the first time—demonstrated how sensitive the methods might be when providing a prognosis. The pathological findings and their changes were capable of predicting good/poor prognosis. In the case of EEGs, normal findings in all of the three examinations were a strong predictor of good outcome, and worsening/improvement might predict poorer/better outcome later. As for TMS examinations, the examination on day 14 was the best prognostic factor. Nevertheless, if we examined patients who were dead at 3 months and had neurophysiological examinations, all patients had EEG abnormalities (diffuse slowing on admission or worsening as defined in *Methods*) and at the same time absence of MEPs or MEP with reduced amplitude. Multivariate analysis was also done, but because of the small numbers within the groups and too many variables, no significant predictor was found [Exp (*B*) 0.750, *p* = 0.594], and therefore, the analysis would lead to misinterpretation. Despite this fact, our findings have the value of real-life scenario, and consecutive patients with ICH have been enrolled with a relatively high sample size using complex clinical, imaging, and neurophysiological examinations.

**Table 5:** Predictors of on-admission disability (mRS) and outcome (mRS at discharge, mRS at 3 months) with univariate analysis [not significant (NS)], unless significant parameters are listed in at least one column.

**Parameter**	**mRS on admission**	**mRS at discharge**	**mRS at 3 months**
Age (>60 years)	NS	0.021	NS
Previous ischemic stroke	NS	NS	0.021
On-admission INR >1.7	NS	NS	0.045
Glasgow Coma Scale score	0.012	0.005	<0.001
Localization (basal ganglia + thalamus and pons mesencephalon—worse)	0.001	0.006	<0.001
Intraventricular and subarachnoidal appearance	0.002	0.05	0.015
Blood in the prepontine cistern	NS	0.045	NS
Volume of bleeding on CT	0.001	0.009	0.001
Volume of edema on CT	0.001	0.05	0.013
Volume of bleeding + edema on CT	0.001	0.002	0.001
Pyramidal tract affected as shown by CT	0.01	0.019	NS
TMS abnormalities	NA	<0.001	<0.001
EEG worsening at 14 days	NA	0.001	0.005
EEG worsening from 14 days to 3 months	NA	0.008	0.057
EEG pathology unilateral/bilateral	NA	<0.001	<0.001
EEG pathological findings	NA	<0.001	<0.001
Mannitol	NA	0.003	<0.001

*Mean discharge time was 19.8 days (1;79)*.

## Discussion

Stroke, especially hemorrhagic stroke, leads to death and severe disability in a high proportion of patients. Therefore, methods that may predict the outcome play an important role in daily clinical routine. Most studies in this field investigate clinical and radiographic predictors of ICH outcome, for example, age, premorbid functional state, initial GCS score, blood pressure, hematoma location, and volume (3, 4, 19, 28–30). So far, many factors are known, but little has been published about the importance of neurophysiological examinations such as EEG and evoked potentials, which give information about function and prognosis, and at which time point it is most relevant. In our prospective study, we looked for factors that, among them neurophysiological parameters, together with imaging and clinical findings, may predict the outcome at 14 days or at discharge and at 3 months.

### Baseline Characteristics and Admission Parameters

There were significantly (*p* < 0.05) more male patients in our database. It is also important to note that—compared to females—male patients were younger on average (mean difference was 4.8 years). Our previous studies among ischemic stroke patients have also shown male predominance, and they have had more severe stroke, at a younger age. This shows that their health condition is generally worse. This might mean that being a male patient means a higher risk for stroke (31, 32). Hypertension is a known risk factor for ICH, which can also be demonstrated by the history of our patients; 89% of them had hypertension, and what is even more important, half of them were not treated for it. Especially their systolic blood pressure was high; the diastolic values were mostly normal or only mildly elevated. Untreated hypertension and high systolic blood pressure on admission are more commonly found in this study than in certain previous databases (32, 33). These facts emphasize the importance of primary screening and education among laypeople. By means of screening and education, some of the tragic ICH cases could have been prevented. Although blood sugar levels were not fasting values, in one in five patients (21.5%), the serum glucose concentration exceeded 10 mmol/L. The prevalence of ischemic stroke was similar to previous data, but previous hemorrhagic stroke was rare (27, 28), and 12.9% of the patients received chronic anticoagulation treatment, whereas 19.8% was given antiplatelet drug. Among the patients, 12.9% had atrial fibrillation, but heart failure was rare. These data are similar to our findings with ischemic stroke patients (31–33).

### Neurological Findings and Imaging

One of the important clinical signs influencing the outcome and mortality in ICH patients is the disturbance of consciousness (2, 3, 5). On admission, more than half of our patients had only mild impairment or none, but with univariate analysis, it was a significant prognostic factor of outcome.

On admission, the enrolled patients had moderate and severe symptoms based on the NIHSS score and mRS, respectively, which alone might indicate a poor prognosis. High hospital (39.1%) and 3 month mortality figures (46.5%) have been similar to those in recently published studies (4, 5).

ICH was more frequent in the left hemisphere (56%). The size and site of the bleeding influenced the prognosis (3, 5). Worse prognosis was noted, when the bleeding destroyed both the basal ganglia and thalamus or pons, or the midbrain.

As expected, on-admission bleeding volume gradually decreased over 14 days and 3 months. Contrarily, the edema volume increased over 14 days, and then in 3 months, it reached the value as in the first CT scan. We found that the bleeding volume together with the volume of the perihematomal edema was high not only on admission, but also at 14 days, and might even have caused a midline shift. This combined volume seemed to be a better predictor according to our neurophysiological examinations, where the on-admission normal findings or mild abnormalities worsened for 14 days but improved at 3 months. With univariate analysis, the blood and edema volume and their combined sum all had a significant effect on the outcomes (Table 5). Gebel et al. (7) investigated the absolute and relative edema (edema volume divided by hematoma volume). They found that only relative edema was strongly predictive of functional outcome.

Almost 50% of the patients had intraventricular and subarachnoid appearance, which decreased relatively quickly and was predictive of a poor outcome. Similarly, intraventricular bleeding was a risk for worse prognosis in the literature (3, 5).

IVH extension after spontaneous supratentorial ICH is a risk of worse outcome and neurological deterioration (34), resulting in death in 32 to 43% (35).

The volume of the IVH alone is not always informative; sometimes even a smaller amount of blood in the intraventicular space can cause severe symptoms, and higher amount less significant (e.g., occlusion of the foramens or the third and fourth ventricles contains the majority of the bleeding and disturbs the cerebrospinal fluid's circulation, leading to extended ventricles).

In the literature, some useful quantitative and semiquantitative methods can be found measuring IVH extension (27) and modified Graeb Scale (35), but measuring IVH volume is possible too (36).

Compared to previous semiquantitative examinations, in our study similar results were found for early outcome of brain hemorrhage, but our sample size of patients having IVH at 14 days and 3 months was very small for detailed statistical analysis. Despite we did not give scores in case of Graeb Scale, we determined the localization similarly to it. Altogether our results show that the intraventricular presence of blood is a bad prognostic value. The involvement of the pyramidal tract means more severe symptoms on admission and at 14 days, just like in TMS examinations, while improvement or worsening at 3 months may be of interest when making a prognosis.

### Neurophysiological Examinations and Outcome

On admission, most of our patients were severely disabled (mRS 5), but by day 14 and over 3 months, their disabilities improved, although mortality was high, but the independent favorable (mRS 0–2) patients' number increased within the survivor group. The mortality rate was different in the different examinations, but mostly similar to our findings (3–5, 37).

By comparing the groups with and without neurophysiological examinations, it can be concluded that the patients in the second group were in a more severe clinical condition, and their decline was very pronounced (NIHSSS, mRS, both) among them. They had severe clinical symptoms and were in an unstable condition to be transferred to time-consuming examinations. Nevertheless, we have studied the prognosis among the patients within the EEG/TMS group (74 on admission) and compared the findings within the group. Unfortunately, many of them died as well, but ICH often has a fatal outcome. Partially, this was the reason why not all ICH patients were studied with combined neurophysiological examinations, and second, follow-up examinations could not always be performed because of the pandemic. Nevertheless, in our center, consecutively every patient was examined, and followed if possible, so we have data about them even if those data do not always meet the requirements of an international multicentric study. Kimiskidis assumed that TMS coupled with EEG might be a biomarker of the future in patients with epilepsy (38). In the literature, the prognostic value of EEG and MEP in ICH outcomes has not been extensively studied, especially during long follow-up period, showing the difficulties of the complex examinations in brain hemorrhage.

### EEG Examination

In the early stages, localized polymorphic delta wave activity appeared ipsilateral in patients with ICH without a shift of midline structures, regardless of the location of hematoma. In patients with hematomas of 30 cm^3^ or larger, causing a shift of the midline structures, delta wave activity appeared over both hemispheres. At the same time, superposition of faster frequencies on slow waves indicated the development of edema in the brain (9).

We found that slow rhythm and theta and delta frequency (of high amplitude) were the most common pathological patterns on the EEG, independently of the location/site of bleeding. Interestingly, amplitude reduction was more common in case of bleedings into lobar and basal ganglia, and we could detect epileptiform discharges in these locations (3.9 %), whereas in the study by Woo, it ranged from 2.8 to 18.7% (10). In our study, only one patient had a focal clinical seizure; we started carbamazepine treatment. Observation of the epileptiform discharges (detected by EEG or during early seizures) is very important, because they might be associated with the deterioration of mental functions or later developing epileptic seizures; therefore, the observation of these patients is advised (6, 39).

Bilateral pathological EEG patterns were detected mostly in bleeding into the basal ganglia plus thalamus simultaneously and bleeding into the thalamus alone. This emphasizes the importance of corticothalamic and thalamocortic pathways (28). Only unilateral pathological patterns were detected in lobar bleedings. These are important from the point of view of prognosis, as the bilateral pathologic changes of EEG correlated with significantly worse outcome with univariate analysis and multivariate analysis (at discharge). Slow rhythm activity was characteristic in EEG pattern on admission and at 14 days in patients who died before the examination at 3 months (mRS was 6) or mRS score was 3 to 5. When the EEG was normal, good outcome (0–2 mRS score) was detected. The development of bilateral EEG abnormality in case of unilateral bleeding is a complex process. In case of large hemispherical bleedings, mass effect and midline shift may cause the compression of the contralateral hemisphere, causing bilateral abnormal slow rhythm with or without amplitude reduction in the EEG pattern. In small unilateral hemispherical hemorrhage, especially in case of combined bleeding into the basal ganglia and thalamus and bleeding into the thalamus alone, bilateral EEG abnormality may develop because of diaschisis effect, which is an abnormal functional disturbance. The primary mechanism of diaschisis is functional deafferentation, which is the loss of the input of information from the part of the brain that is now damaged (40). This is a possible explanation of bilateral EEG abnormalities in striatum and thalamus bleedings.

### TMS Examination

Damage to the corticospinal tract can be examined with MEPs elicited by TMS (41–43).

In TMS findings during the examined period, responses most commonly could not be provoked on the affected side. However, if there was any response, low amplitudes were measured. The pathological amplitude (reduction or loss) was the most prominent sign of the prognosis. Escudero et al. (25) performed study of patients with acute ischemic stroke and motor deficit to evaluate the early prognostic value of TMS in motor and functional recovery. According to their results, patients by whom MEP was not detected had worse prognosis, than those who had. They found that the outcome was worse in patients with delayed CCT. Interestingly, in our ICH patients, the amplitude reduction or loss of MEP was the prominent sign. In acute ischemic stroke patients, MEP obtained by TMS represented a useful early prognostic marker of motor function recovery in ischemic stroke patients, and the technique could be used for monitoring and quantifying motor function, in parallel with corticospinal tract permeability, in the course of patient recovery (25). Rossini et al. published also that an absence of MEP indicates poor recovery and higher rate of mortality (44). Our findings concerning brain hemorrhage also support their idea.

Regarding the time when TMS examination was the most helpful for prognosis, there is contradiction. Absence of MEPs obtained during the first week did not necessarily indicate poor recovery at 3 and 6 months later according to Arac et al. (45). Subsequent studies examined the predictability of MEP for motor outcome in stroke patients (41–43). Kwon et al. (43) analyzed the MEP characteristics within 15 days and 16 to 30 days from stroke onset in patients with putaminal bleeding (43). They concluded that the predictability of motor outcome might be better when MEP is performed late than early. Based on the TMS examination on day 14, we could select a group of patients who had a better chance for a good outcome. This was an argument for planning long-term rehabilitation.

In 6% of the patients, despite the possible pyramidal tract lesion on the CT, normal MEP findings with TMS were detected. A possible explanation might be that the intracranial part of the pyramidal tract was just pushed aside by the volume of the bleeding or edema in these cases.

All patients who passed away by month 3 had severe EEG and abnormal MEPs (reduced or absent), which might show the prognostic value of combined neurophysiological examinations.

## Summary

Our work was done in the frame of GINOP IRONHEART study, and in this study, we wanted to find predictors of disability on admission, at 14 days and at 3 months considering different variables. Age was only significant on discharge, and previous ischemic stroke at 3 months. Localization of the bleeding was an important predictor, similarly to the presence of blood in the subarachnoid space and cerebral ventricles. Bleeding, edema, and their combined volume all altered the disability significantly.

To our knowledge, this is the first study in which two neurophysiological modalities (EEG, TMS) were examined within the same study on ICH patients, in order to find out how sensitive the methods might be for providing a prognosis. The pathological findings and their changes could predict good/poor prognosis. In case of EEG, normal results in all of the three examinations are strong predictors of a good outcome. Accordingly, worsening/improvement may be suggestive of a worse/better outcome later. In case of TMS examinations, the ones at 14 days were the best prognostic factors.

In conclusion, in the functional outcome of patients having suffered ICH, the volume of bleeding, perihematomal edema (or their combined volume), and functional neurophysiological examinations, for example, EEG and TMS, play an important role, in addition to the clinical symptoms. This might affect the patients' rehabilitation plans in the future, as with the help of the examinations a potentially recovering group could be selected.

## Limitations

The authors are aware that the study has several limitations. Nevertheless, the strength of the study is the prospective design and the multidisciplinary approach combining imaging techniques, neurophysiological examinations, and clinical findings. However, the number of patients is limited, especially of those in the follow-up. This is partially due to the severe outcomes, which means nursing homes for the patients, and partially to the SARS-CoV-2 pandemic (some of our patients have not been allowed to come from social care homes, and some have been afraid to). Neurophysiological examinations could not be carried out in all patients, which resulted in a selection bias. The above might imply that the study was underpowered to identify all clinically important predictors of outcomes after ICH.

## Data Availability Statement

The raw data supporting the conclusions of this article will be made available by the authors, without undue reservation.

## Ethics Statement

The studies involving human participants were reviewed and approved by Ethics Committee of University of Debrecen and the Ethics Committee of the National Medical Research Council. The patients/participants provided their written informed consent to participate in this study.

## Author Contributions

KF and IF led the initiative, selected the abstract, extracted data, and drafted the manuscript. SM, LH, KF, and IF were involved in data analysis and writing the original draft. MH, TÁ, KF, and IF were involved in data collection. JT was responsible for imaging data collection and analysis. LC and ZB were involved in the supervision of manuscript drafting. DS, KF, and IF were involved in neurophysiological workup. All authors were involved of the conceptualization and editing. All authors approved the final version of the manuscript.

## Funding

This study is supported by grants from the National Research, Development and Innovation Fund (K109712, K120042, and FK128582), by GINOP-2.3.2-15-2016-00043 and the Hungarian Academy of Sciences (MTA-DE Cerebrovascular and Neurodegenerative Research Group).

## Conflict of Interest

The authors declare that the research was conducted in the absence of any commercial or financial relationships that could be construed as a potential conflict of interest.

## Publisher's Note

All claims expressed in this article are solely those of the authors and do not necessarily represent those of their affiliated organizations, or those of the publisher, the editors and the reviewers. Any product that may be evaluated in this article, or claim that may be made by its manufacturer, is not guaranteed or endorsed by the publisher.
